# Iodoform in Nasal Catarrh and the Treatment of Wounds

**Published:** 1880-08

**Authors:** 


Iodoform in Nasal Catarrh and the Treatment of Wounds.—Dr. Albert Wilson finds that iodoform is very useful in cases of post-nasal catarrh. He has tried the remedy in a large number of cases with great success. The coryza and accompanying pain and discomfort disappeared in a few hours, or in the course of a night. In cases of a mild catarrh, however, iodoform aggravated the symptoms. The method of treatment was to snuff up each nostril about as much of the powder as would lie upon a threepenny piece. One application is enough.

Iodoform has also been used with success in the treatment of wounds. It is especially useful in strumous subjects, when the wounds do not seem inclined to heal. In antiseptic cases, the powder is to be mixed with a carbolic acid solution of strength one in forty, to render inert atmospheric dust which may be mixed with it. It is also very useful in cases where there are sloughs; the sloughs rapidly disappear, and the wound becomes clean and healthy. Dr. Hack has recently shown the absorbing power of granulations, and that they will absorb aseptic sloughs. This latter fact was originally pointed out by Prof. Lister, not only in regard to aseptic sloughs, but also in some cases to aseptic necrosed bone. The iodoform is probably absorbed by the granulation, stimulating them to absorb the slough much more rapidly. In these cases Dr. Wilson had noticed that the sloughs disappear without alteration in their external surfaces, and without increased discharge. They are in fact absorbed from the under surfaces, which lie in contact with the granulations. In painful burns an ointment containing iodoform soothes the pain, whilst the powder sprinkled on gives very great pain. The author concludes by suggesting that iodoform may be of use in obstinate cases of photophobia, employed either as an ointment spread along the lids, or as a snuff.—*Brit. Med. Journal.*

